# Clinical Efficacy of Jinshuibao Capsules Combined with Angiotensin Receptor Blockers in Patients with Early Diabetic Nephropathy: A Meta-Analysis of Randomized Controlled Trials

**DOI:** 10.1155/2018/6806943

**Published:** 2018-04-24

**Authors:** Qiang Lu, Cailan Li, Weiwen Chen, Zhongfeng Shi, Ruoting Zhan, Rui He

**Affiliations:** ^1^Key Laboratory of Chinese Medicinal Resource from Lingnan, Ministry of Education and Research Center of Chinese Herbal Resource Science and Engineering, Guangzhou University of Chinese Medicine, Guangzhou 510006, China; ^2^Guangdong Provincial Key Laboratory of New Drug Development and Research of Chinese Medicine, Mathematical Engineering Academy of Chinese Medicine, Guangzhou University of Chinese Medicine, Guangzhou 510006, China; ^3^Center Laboratory, Guangdong Pharmaceutical University, Guangzhou 510006, China

## Abstract

**Background:**

Jinshuibao capsules (JSB) have been widely used to treat early diabetic nephropathy (DN), but the specific effects are still inconsistent. A meta-analysis of randomized controlled trials (RCTs) was conducted to evaluate the clinical efficacy of JSB for early DN.

**Methods:**

Four international databases and four Chinese databases were searched from publication dates to March 1, 2018. The RCTs reporting the results of JSB's specific effects were included, and comparisons were between JSB combined with Angiotensin Receptor Blockers (ARBs) as experimental intervention and ARBs as the control. Included studies' quality was evaluated and the extracted data were analyzed with RevMan 5.3 software.

**Results:**

Twenty-six RCTs including 2198 early DN participants were adopted in the meta-analysis. The results showed that, compared with the ARBs alone, JSB could remarkably improve the ORR (OR = 3.84; 95% CI: 2.37~6.24; *P* < 0.00001) and decrease 24 h UTP (MD = −93.32; 95% CI: −128.60 ~−58.04; *P* < 0.00001), UAER (MD = −24.02; 95% CI: −30.93 ~−17.11; *P* < 0.00001), BUN (MD = −0.26; 95%: −0.44 ~−0.08; *P* = 0.005), Scr (MD = −9.07; 95% CI: −14.26 ~−3.88; *P* = 0.0006), ACR (MD = −17.55; 95% CI: −22.81 ~−12.29; *P* < 0.00001), Cys-C (MD = −0.60; 95% CI: −0.88 ~−0.32; *P* < 0.00001), SBP (MD = −3.08; 95% CI: −4.65 ~−1.52; *P* = 0.0001), DBP (MD = −2.09; 95% CI: −4.00 ~−0.19; *P* = 0.03), and TG (MD = −0.36; 95% CI: −0.50 ~−0.21; *P* < 0.00001). However, it showed no significant differences in TC (MD = −0.32; 95% CI: −0.69~0.04; *P* = 0.08), FBG (MD = 0.04; 95% CI: −0.39~0.47; *P* = 0.87), HbA_1c_ (MD = −0.26; 95% CI: −0.59~0.06; *P* = 0.11), and *β*_2_-MG (MD = −15.61; 95% CI: −32.95~1.73; *P* = 0.08).

**Conclusions:**

This study indicates that JSB is an effective accessory therapeutic medicine for patients with early DN. It contributes to decreasing blood pressure and the content of triglyceride and improving the renal function of early DN patients. However, there is still a need to further verify the auxiliary therapeutic effect of JSB with more strictly designed RCTs with large sample and multiple centers in the future.

## 1. Introduction

As one of the common and severe microvascular complications of diabetes mellitus (DM), DN is getting much more attention [1, 2]. DN is a type of kidney damage caused by DM. Its pathogenesis is closely linked to many factors, mainly including glucose metabolic disorder, hemodynamic abnormality, and oxidative stress [3]. Once developed into end-stage kidney disease, the treatment of DN would be more difficult than other kidney diseases for the complex metabolic disorders [4]. Therefore, timely prevention and treatment for DN will become more urgent.

Based on Mogensen Stage, DN can be divided into five stages: stage I, high perfusion or kidney hypertrophy; stage II, normal urinary albumin excretion rate (UAER); stage III, also called early DN, microalbumin appearing in the urine; stage IV, also called clinical or dominant DN, plenty of albumin appearing in the urine; and stage V, end-stage renal disease (ESRD) [5, 6]. In order to prevent entry into the ESRD phase, therapeutic measures must be adopted in early stages of DN. While the disease symptoms in stages I and II of DN are unconspicuous, most patients with DN were found in stage III or after stage III [7]. And when DN enters stage Ⅳ, the reactivity of patients to drugs become deteriorated and the improvement and maintenance of the condition becomes more difficult [8]. In consequence, based on these problems, the prevention and treatment for DN in stage III (i.e., early DN) would be comparatively reasonable and very important.

A large number of studies about early DN had been carried out, and some progress has been achieved in the understanding and treatment of early DN. Currently, on the basis of DM treatments including strict control of blood pressure and blood sugar and attention to diet and moderate exercise, ARBs combined with Chinese traditional medicine and ARBs alone are the main treatment method for early DN [35]. Many clinical studies showed that the method of therapy of ARBs combined with Chinese traditional medicine showed some advantages in many aspects, such as enhancing efficacy, decreasing adverse reactions, and reducing toxicity, compared to ARBs alone [36].

Traditional Chinese medicine (TCM) adopts a typical symptoms-based method, with history-proven treatment effect [37]. Jinshuibao capsule, produced by Jimin kexin pharmaceutical company, is the first approved new drug belonging to Category 1 of Traditional Chinese Medicines, since Ministry of Health in China has formulated provision for new drug approval [38].* Cordyceps sinensis* isolated from fresh Chinese caterpillar fungus in Qinghai, experienced purification and fermental cultivation and was finally processed into JSB [39]. JSB has been applied to clinical treatment for a long time and has been proved to hold good effects on the treatment of many diseases, such as chronic nephritis, pulmonary tuberculosis, and diabetic nephropathy [40]. While there are many clinical researches on JSB in treating early DN, the related evidence is still inconsistent and not systematic. Therefore, we made a meta-analysis of RCTs, to determine whether or not JSB is beneficial to patients with early DN and what aspects JSB improved.

Due to the extensive use of JSB, participants were brought into the study regardless of some individual characteristics, such age or sex. JSB combined with ARBs was used in experimental groups, and ARBs alone were administered in control groups. Outcomes contained some indicators about renal function, some DM related indicators, and adverse reaction.

## 2. Methods

### 2.1. Search Strategy

For this meta-analysis, the related international databases were selected and searched: PUBMED, EMBASE, Cochrane Library, BMJ Clinical Evidence, and International Clinical Trials Registry Platform. And the related Chinese databases were also selected and searched: China National Knowledge Infrastructure database (CNKI), Chinese Biomedical Literature database (CBM), Wanfang database, and VIP database. The retrieval task was conducted by Lu Qiang and Li Cailan, and the retrieval time is from inception to November 20, 2017. Two different retrieval strategies were adopted as follows: the retrieval terms “Jinshuibao/JSB” and “diabetic nephropathy/diabetic nephrosis/DN/diabetic kidney disease/DKD” were adopted in the English databases; the searching terms “Jinshuibao (in Chinese)” and “tang niao bing shen bing (which means nephrosis in Chinese)” were used in the Chinese databases. Only Chinese literature and English literature were searched, and the animal experiments were removed.

### 2.2. Inclusion and Exclusion Criteria

Inclusion criteria were as follows: (1) the study was conducted as a RCT; (2) patients were diagnosed with DM by the diagnostic standard of WHO and early DN by the staging criteria of Mogensen; (3) the experimental group was given the combined treatment of JSB and ARBs, and ARBs alone as the control; (4) the studies reported one or some related outcomes of DN, such as the overall response rate (ORR), the content of 24-hour urinary total protein (24 h UTP), urine albumin excretion ratio (UAER), blood urea nitrogen (BUN), serum creatinine (Scr), albumin-to-creatinine ratio (ACR), cystatin C (Cys-C), *β*_2_-microglobulin (*β*_2_-MG), fasting blood glucose (FBG), hemoglobin A_1c_ (HbA_1c_), systolic blood pressure (SBP), diastolic blood pressure (DBP), serum total cholesterol (TC), and triglyceride (TG); (5) remedy continued for 8 weeks or longer. Exclusion criteria were the following: (1) other stages of diabetic nephropathy; (2) duplication in the clinical data with the same authors, but published in different periodicals; (3) diagnostic criteria, intervention measures, or outcome indicators not being clarified clearly or being not appropriate; (4) inability to get the full text.

### 2.3. Data Extraction

Detailed data extracted from the studies included author's name, publication date, number of patients, age of participants, sex, disease course, treatment course, details of intervention, and relevant outcome indicators. Selected data were used to conduct statistical analysis, in which drop-outs were regarded as treatment failures in combining therapy groups, contrary to the control groups. The Cochrane Collaboration tool was used to assess the methodological quality. In order to avoid bias, eligibility evaluation of searched literature, study selection, data abstraction, and assessment of study quality were conducted, respectively, by Lu Qiang and Li Cailan, based on the standards of Cochrane Handbook. Studies were screened and extracted data were checked several times to ensure internal consistency. Discussion with Zhan Ruoting and He Rui was put forward if there are differences.

### 2.4. Statistical Analysis

The official software RevMan 5.3 of the Cochrane Collaboration was applied to conduct the meta-analysis. The integral heterogeneity among adopted studies was evaluated by *I*^2^ test and *Z*-test analysis. *I*^2^ ≤ 50% or *P* ≥ 0.10 means that the heterogeneity is not significant and the results can be pooled to be calculated with the fixed effect model; if not, the random effect model was selected [41]. The confidence interval (CI) was set to 95%, and continuous data were presented as mean differences (MD) [42]. The main focus of this study was some outcome indicators associated with early DN (ORR, 24 h UTP, UAER, ACR, BUN, Scr, Cys-C, *β*_2_-MG, FBG, HbA_1c_, SBP, DBP, TC, and TG). The potential publication bias of the included studies was assessed by a funnel plot.

## 3. Results

### 3.1. Brief of Accepted Studies

**Figure 1:**
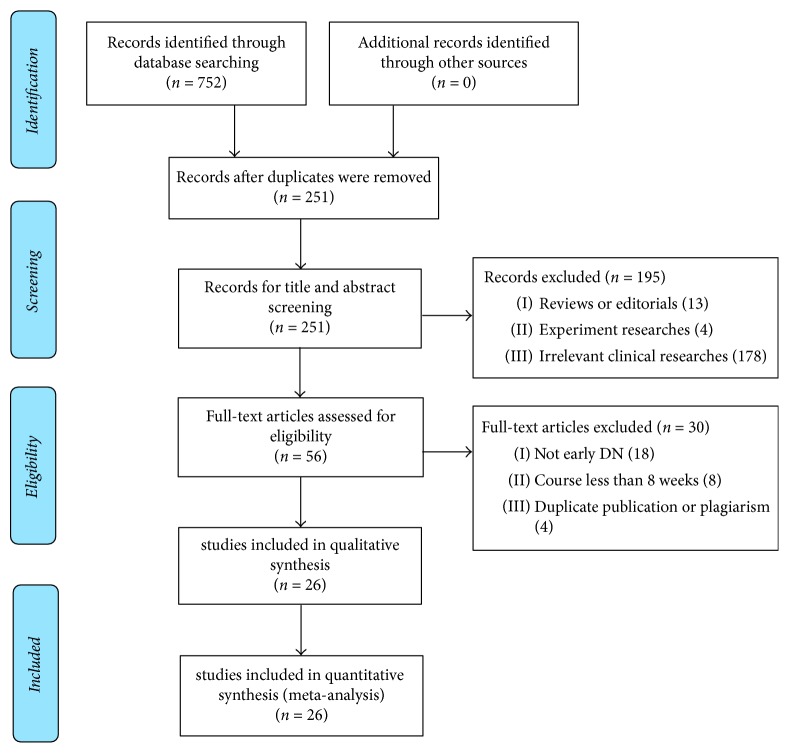
Flowchart of screening eligible studies about JSB and early DN.

A total of 752 studies were found with both cyber and manual retrieval of cited references, in which 501 repetitions were detected, 195 studies were removed for improper titles and abstracts, and 30 studies were excluded after full-text reading. Finally, 26 studies met the inclusion requirement for meta-analysis. The flowchart describing the process of selecting studies was displayed in Figure 1. All 26 studies were carried out in China. The number of participants in each study differed from 40 to 205, with a total of 2198 patients included. A suitable description for the basic characteristics of the included studies was showed in Table 1. JSB was given in doses from 3 to 6 capsules three times a day based on age. ARBs depended on age and weight according to the drug use instructions.

### 3.2. Methodological Quality of Included Studies

**Figure 2:**
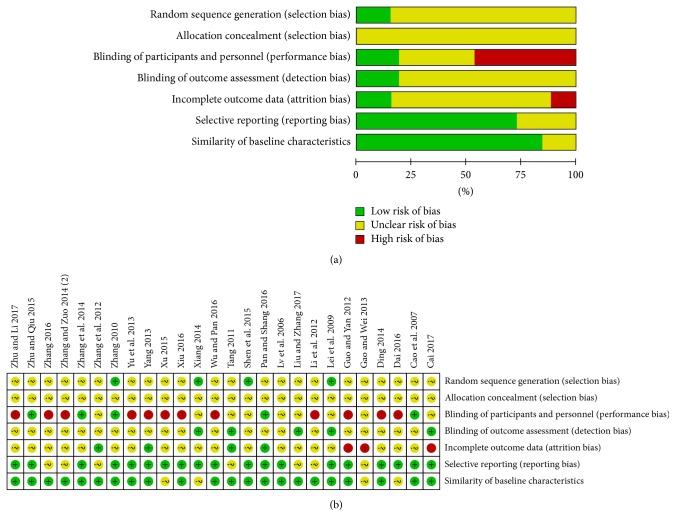
The evaluation for the risk of bias with Review Manager 5.3. (a) Risk of bias graph; (b) risk of bias summary.

The risk of bias evaluation for the included studies is shown in Figure 2. Risk of bias was found across studies from seven aspects including random sequence generation, allocation concealment, blinding of participants and personnel, blinding of outcome assessment, incomplete outcome data, selective reporting, and similarity of baseline characteristics. Randomization was mentioned among all studies, but only four studies described the random sequence generation detailedly. Allocation concealment was not discussed in all studies. Five studies made a description about blinding of patients and personnel, while other studies did not. Few studies stated the details about blinding of outcome assessment.

### 3.3. Effect of JSB on ORR

**Figure 3:**
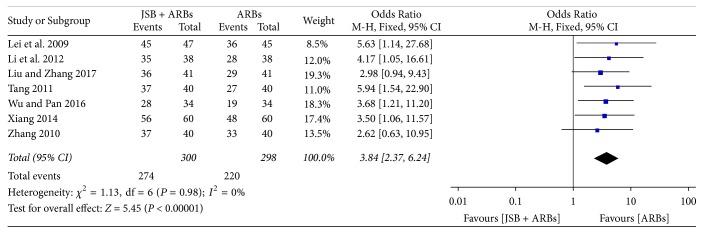
Forest plot of RCTs reporting the effect of JSB on ORR.

Seven [11–13, 15, 21, 29, 33] of 26 studies compared the ORR between JSB combined with ARBs therapy and ARBs therapy. There was no heterogeneity (*P* = 0.98, *I*^2^ = 0%), and a fixed effect model was applied to conduct the meta-analysis. An OR with 95% CI was used to present the comparison of the ORR between the experimental and control groups (OR = 3.84; 95% CI: 2.37~6.24; *P* < 0.00001). It indicated that JSB could significantly improve the therapeutic effect of ARBs for early DN (Figure 3).

### 3.4. Effect of JSB on Renal Functions

We analyzed the RCTs that measured 24 h UTP, UAER, BUN, Scr, ACR, Cys-C, and *β*_2_-MG.

#### 3.4.1. Effect of JSB on 24 h UTP

**Figure 4:**
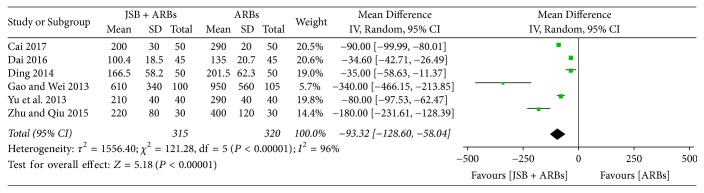
Forest plot of RCTs reporting the effect of JSB on 24 h UTP.

There were six trials [17, 19, 20, 26, 27, 32] reporting the results about 24 h UTP before and after treatment. As the pooled results showed statistical heterogeneity (*P* < 0.00001, *I*^2^ = 96%), a random effect model was adopted for data analysis. Forest plot's results (Figure 4) showed that the MD was −93.32 (95% CI: −128.60~−58.04; *P* < 0.00001), indicating that JSB greatly contributed to decreasing the content of 24 h UTP in patients with early DN.

#### 3.4.2. Effect of JSB on UAER

**Figure 5:**
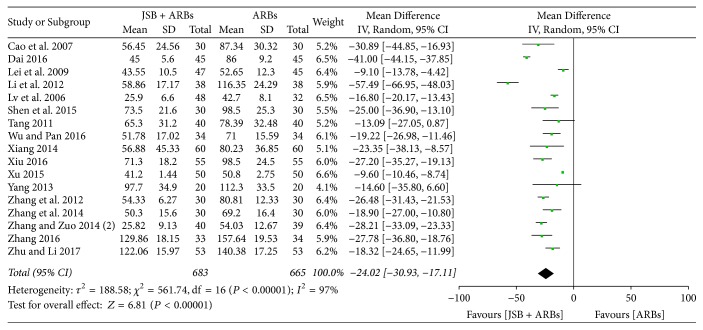
Forest plot of RCTs reporting the effect of JSB on UAER.

Seventeen studies [9–11, 13, 15, 16, 18, 21–25, 27, 29–31, 34] reported UAER in the experimental group and control group. The forest plot showed poor homogeneity (*P* < 0.00001, *I*^2^ = 97%), and a random effect model was applied for data analysis. As shown in Figure 5, JSB + ARBs had a greater advantage of decreasing UAER than the ARBs group (MD = −24.02; 95% CI: −30.93~−17.11; *P* < 0.00001).

#### 3.4.3. Effect of JSB on BUN

**Figure 6:**
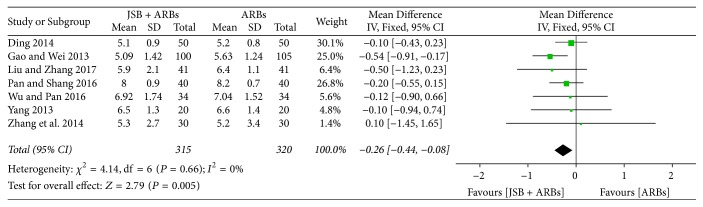
Forest plot of RCTs reporting the effect of JSB on BUN.

Seven studies [17, 18, 20, 22, 28, 29, 33] reported the data on BUN in JSB + ARBs group and ARBs group. There was no heterogeneity from these studies to be found (*P* = 0.66, *I*^2^ = 0%), so a fixed effect model was selected to conduct data analysis. As shown in Figure 6, JSB had a certain effect on the content of BUN in early DN patients (MD = −0.26; 95%: −0.44~−0.08; *P* = 0.005).

#### 3.4.4. Effect of JSB on Scr

**Figure 7:**
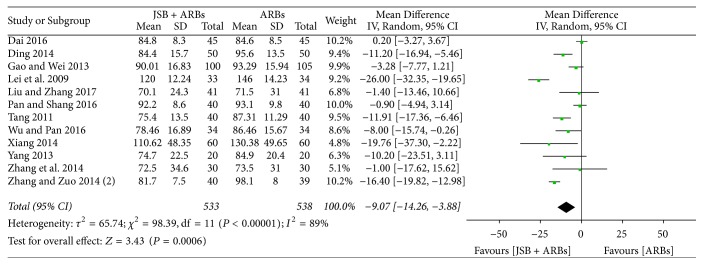
Forest plot of RCTs reporting the effect of JSB on Scr.

There were twelve researches [11, 13, 17, 18, 20–23, 27–29, 33] incorporated in the meta-analysis of decline of Scr. Obvious heterogeneity was discovered among Scr data from the accepted researches (*P* < 0.00001, *I*^2^ = 89%), so a random effect model was selected to conduct data analysis. As displayed in Figure 7, the level of Scr was significantly reduced in the treatment with JSB + ARBs compared with ARBs group, indicating that JSB helps to decrease the content of Scr (MD = −9.07; 95% CI: −14.26~−3.88; *P* = 0.0006).

#### 3.4.5. Effect of JSB on ACR

**Figure 8:**
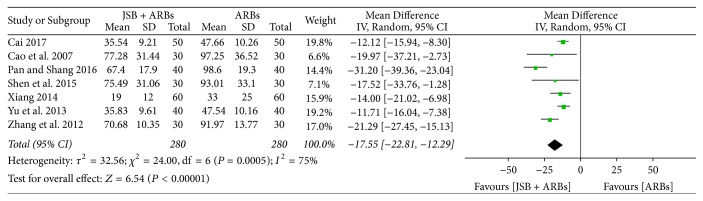
Forest plot of RCTs reporting the effect of JSB on ACR.

Seven studies [10, 16, 19, 21, 24, 28, 32] reported the ratio of ACR at the end of the treatment. The forest plot showed evident homogeneity (*P* = 0.0005, *I*^2^ = 75%), so a random effect model was selected for data analysis. As shown in Figure 8, JSB combined with ARBs had an advantage of decreasing the ratio of ACR compared to the ARBs group (MD = −17.55; 95% CI: −22.81~−12.29; *P* < 0.00001).

#### 3.4.6. Effect of JSB on Cys-C

**Figure 9:**
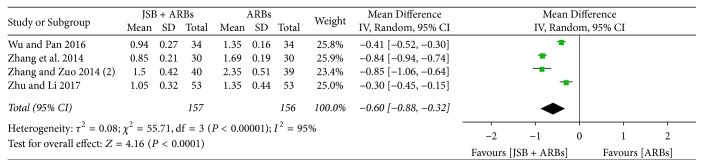
Forest plot of RCTs reporting the effect of JSB on Cys-C.

Five studies [22, 23, 29, 34] reported the concentration of cystatin C (Cys-C) in the experimental and control groups. The pooled data were shown to be homogeneous (*P* < 0.00001, *I*^2^ = 95%), and a random effect model was used for meta-analysis. The forest plot's results (Figure 9) showed that JSB has an additional effect on reducing the concentration of Cys-C in early DN patients (MD = −0.60; 95% CI: −0.88~−0.32; *P* < 0.00001).

#### 3.4.7. Effect of JSB on *β*_2_-MG

**Figure 10:**
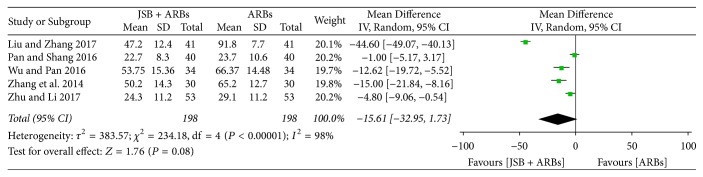
Forest plot of RCTs reporting the effect of JSB on *β*_2_-MG.

Five studies [22, 28, 29, 33, 34] reported the concentration of *β*_2_-microglobulin (*β*_2_-MG) after the treatment cycle. There was great heterogeneity (*P* < 0.00001, *I*^2^ = 98%), and a random effect model was used to perform the meta-analysis. As shown in Figure 10, JSB has no effect on *β*_2_-MG (MD = −15.61; 95% CI: −32.95~1.73; *P* = 0.08).

### 3.5. Effect of JSB on Blood Glucose

We analyzed the RCTs that measured FBG and HbA_1c_.

#### 3.5.1. Effect of JSB on FBG

**Figure 11:**
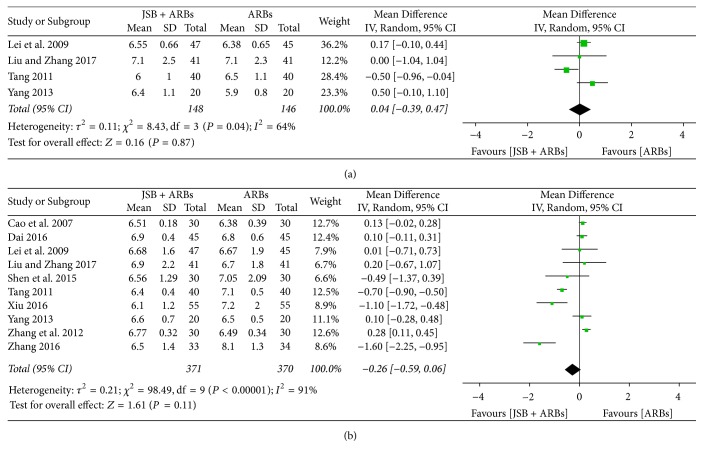
Forest plot of RCTs reporting the effect of JSB on blood glucose. (a) FBG; (b) HbA_1c_.

Four studies [11, 13, 18, 33] reported the concentration of fasting blood glucose (FBG) in the experimental and control groups. There was certain heterogeneity (*P* = 0.04, *I*^2^ = 64%), and a random effect model was used to perform the meta-analysis. As shown in Figure 11(a), JSB has no effect on FBG (MD = 0.04; 95% CI: −0.39~0.47; *P* = 0.87).

#### 3.5.2. Effect of JSB on HbA_*1*c_

Ten studies [10, 11, 13, 16, 18, 24, 27, 30, 31, 33] reported the percentages of HbA_1c_ after therapy. The extracted data were not shown to be homogeneous (*P* < 0.00001, *I*^2^ = 91%), confirming the random effect model applied for data analysis. As shown in Figure 11(b), JSB did not have an additional effect on the HbA_1c_ level in early DN patients (MD = −0.26; 95% CI: −0.59~0.06; *P* = 0.11).

### 3.6. Effect of JSB on Blood Pressure

We analyzed the RCTs that measured SBP and DBP.

#### 3.6.1. Effect of JSB on SBP

**Figure 12:**
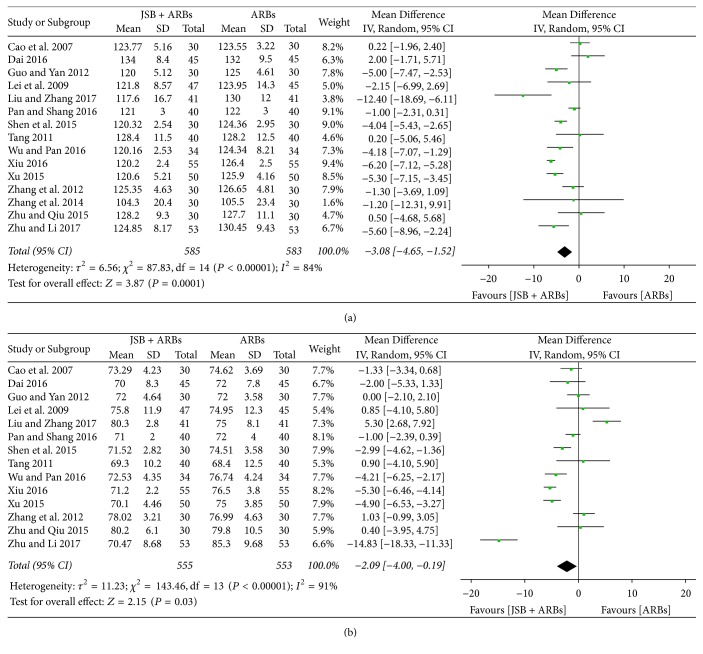
Forest plot of RCTs reporting the effect of JSB on blood pressure. (a) SBP; (b) DBP.

Fifteen studies [10, 11, 13, 14, 16, 22, 24–30, 33, 34] reported SBP of patients after the treatment cycle. The abstracted data showed remarkable heterogeneity (*P* < 0.00001, *I*^2^ = 84%), so the random effect model was applied for data analysis. As shown in Figure 12(a), JSB combination group was more conducive to lower the SBP than the control group (MD = −3.08; 95% CI: −4.65~−1.52; *P* = 0.0001).

#### 3.6.2. Effect of JSB on DBP

Fourteen studies [10, 11, 13, 14, 16, 24–30, 33, 34] reported DBP of patients after the treatment cycle. The extracted data were shown to be homogeneous (*P* < 0.00001, *I*^2^ = 91%), so the random effect model was used for data analysis. As shown in Figure 12(b), JSB + ARBs group has a lower DBP level compared with the control group, indicating that JSB contribute to lowering the DBP level in patients with early DN (MD = −2.09; 95% CI: −4.00~−0.19; *P* = 0.03).

### 3.7. Effect of JSB on Blood Lipid

We analyzed the RCTs that measured TC and TG.

#### 3.7.1. Effect of JSB on TC

**Figure 13:**
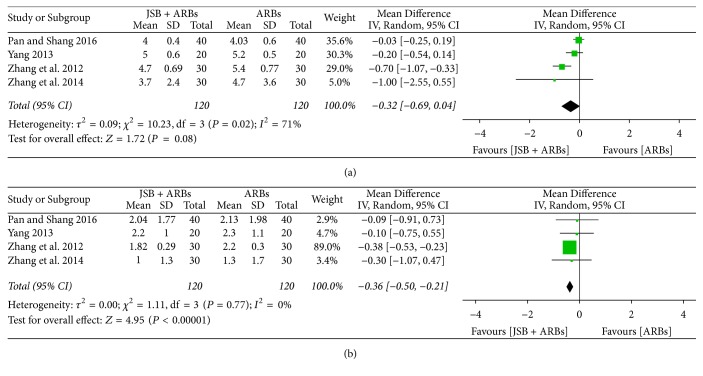
Forest plot of RCTs reporting the effect of JSB on blood lipid. (a) TC; (b) TG.

There were four trials [16, 18, 22, 28] accepted in the meta-analysis reporting the content of Serum TC after treatment. As the pooled results showed statistical heterogeneity (*P* = 0.02, *I*^2^ = 71%), the random effect model was adopted for data analysis. The forest plot's results showed that the MD was −0.32 (95% CI: −0.69~0.04; *P* = 0.08), indicating that there was no significant difference in the content of TC between the two groups (Figure 13(a)).

#### 3.7.2. Effect of JSB on TG

Four studies [16, 18, 22, 28] reported triglyceride (TG) of patients after the treatment cycle. While the extracted data were shown to be homogeneous (*P* = 0.77, *I*^2^ = 0%), the fixed effect model was used for data analysis. As shown in Figure 13(b), JSB + ARBs group contributed to lowering the TG more than the ARBs group (MD = −0.36; 95% CI: −0.50~−0.21; *P* < 0.00001).

### 3.8. Adverse Reaction

Only two studies [9, 26] reported the condition of adverse reaction. One study [9] indicated that no drug-related serious adverse events were observed. Another study [26] pointed out that an adverse reaction (i.e., emesis) occurred in both experimental and control groups, and it was relieved without any special treatment. Therefore, the safety of JSB still needs to be considered cautiously in the future clinical trials.

### 3.9. Publication Bias

**Figure 14:**
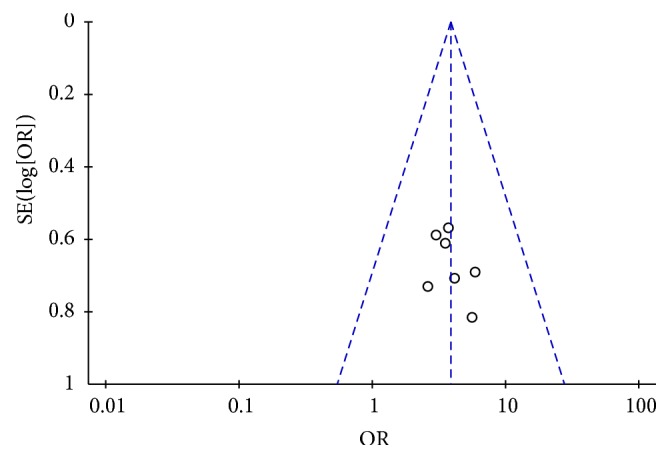
Funnel plot of ORR for the publication bias.

A funnel plot was adopted to explore the publication bias. The publication bias was checked for the ORR. The plot was symmetric, suggesting that there was no obvious publication bias (Figure 14).

## 4. Discussion

DN is one of the major chronic complications of DM and the main reason causing ESRD in the western countries; the proportion is also increasing year by year in China [43]. At present, China has 92 million diabetic patients and 148 million patients with prediabetes [44]. With the increasing number of people with diabetes worldwide, the prevalence of DN also increases. In consequence, it seems to be quite important that DN could be promptly detected and effective measures could be taken. As a severe microvascular complication of DM, the complicated pathogenesis of DN has not been fully elucidated, mainly because a variety of factors join together and affect each other, like metabolic and hemodynamic disorders, oxidative stress, inflammation, and hereditary factor, and so on [45].

JSB is a Chinese patent medicine, jointly researched and developed by Shanghai Institute of Medicine of Chinese Academy of Medical Sciences and Jiang Xi national pharmaceutical company [46]. JSB is mainly made from fresh* Cordyceps sinensis* in Qinghai, which is a traditional Chinese medicine and is used to enhance the body immunity, nourish lung and kidney, stanch and reduce phlegm, and have a significant inhibitory effect on lung cancer and liver cancer [47].

For the past few years, with increasingly deepened understanding for evidence-based medicine, more and more doctors and pharmacists accepted and applied the conclusions of systematic review and meta-analysis to direct their clinical practice [48]. Although most of the relevant clinical researches were carried out in China, and it is difficult to obtain these clinical information about JSB for foreign researchers by online academic databases, the active functions of JSB should not be neglected and underrated all over the world. The results of this meta-analysis showed that JSB was likely to develop the anti-DN role by improving the renal function for early DN sufferers, which is consistent with the classical function of* Cordyceps sinensis* on “nourishing lung and kidney” in China. Compared to single clinical study, this stringent and overall meta-analysis could reach a more accurate and scientific conclusion with respect to JSB.

This meta-analysis is the first attempt to synthesize the clinical data of JSB for early DN. The main character of the accepted studies is that the experimental groups received the combined treatment of JSB and ARBs and the control groups only accept the ARBs treatment, on the basis that both the experimental groups and the control groups received conventional treatments of diabetes, which included controlling blood glucose, blood pressure, and blood lipids and taking moderate exercise. Figure 2 showed the methodological quality of final accepted researches. On the whole, the general characteristics of most studies basically coincide, which can guarantee the reliability of this meta-analysis. However, some defects diminishing the quality existed in the accepted researches. Allocation concealment was not mentioned in all the accepted researches and only several researches reported random sequence generation; therefore, the selection bias may be higher. Only several studies reported details about the blinding of participants and personnel or the blinding of outcome assessment. There were also some merits; that is, we only accepted early DN (stage III) based on Mogensen Stage.

We conducted the meta-analysis to evaluate the assistant clinical effect of JSB in the combined treatment for early DN sufferers. A total of 26 trails providing JSB + ARBs versus ARBs to sufferers of early DN were introduced, of which the experimental group has a total of 1105 patients and the control group has a total of 1093 patients. The pooled result of ORR showed that JSB contributed to improving the therapeutic effect of ARBs for early DN. As to the specific effects of renal protection, the levels of 24 h UTP, UAER, BUN, Scr, ACR, Cys-C, and *β*_2_-MG are relatively common in renal function indexes that they are widely used to detect DN [49]. This meta-analysis indicated that JSB + ARBs contribute to lowering the levels of 24 h UTP, UAER, BUN, Scr, ACR, and Cys-C compared with ARBs alone, suggesting that JSB had an adjunctive therapy for the renal protective effect. In addition, some other outcome indicators including SBP, DBP, and TG were also obviously decreased after the treatment of JSB. However, the obtained results were inconsistent with the results of some researches in that we did not find a significant difference on FBG, HbA_1c_, and TC between the experimental group and the control group.

However, there are some possible limitations worthy of being illustrated in this meta-analysis. First of all, although we adopted a comprehensive retrieval strategy to minimize publication bias, some linguistic biases may exist because of language limitations; that is, we only searched the Chinese and English databases. Secondly, the sample size in most included clinical researches is relatively small and the treatment period in some studies is fairly short. There may be a certain limitation to detect a statistically significant difference between JSB + ARBs group and ARBs group. Thirdly, all the involved patients were Chinese. However, including more varied population sample is necessary and can reach richer and more reliable results. Fourthly, only two of the included studies reported the condition of adverse reaction. Although neither study found obvious adverse drug reactions, the lack of information on this aspect raised a worry about the safety of the combination of JSB and ARBs for early DN. Fifthly, most of the included RCTs did not report detailed methodology, the assessment of efficacy is not standard and strict, and the quality of the study design is also not good enough. The sample size and selection criteria varied for the included studies, so we were unable to conduct a subgroup analysis. Sixthly, although we found that combined treatment has potential advantages, the determination of the statistical heterogeneity in some outcomes is still a question in the study. Generally, it is hard to investigate the heterogeneity in the indicators of continuous variables, especially when the number of the included studies is small. And we failed to find the real sources of the heterogeneity after conducting sensitivity analysis and subgroup analysis. We speculated that the heterogeneity was caused by two or more factors, such as age, sex, disease course, and treatment time. Therefore, RCTs that include good methodological quality, favorable experimental design, and larger sample size are needed to explore the auxiliary therapeutic effects of JSB for early DN in the future.

## 5. Conclusions

This meta-analysis indicates that, compared to the ARBs group, JSB in the combination treatment group contributes to improving the ORR and decreasing 24 h UTP, UAER, BUN, Scr, ACR, Cys-C, SBP, DBP, and TG. However, we do not find a significant difference in FBG, HbA_1c_, TC, and *β*_2_-MG between the experimental group and the control group. Therefore, JSB may be an effective accessory therapeutic medicine for patients with early DN. Nevertheless, some accepted studies possessed poor quality, high risk of bias, and small sample size, and in view of the high statistical heterogeneity in some pooled results, there is still a need to continue to verify the conclusion with the strictly designed RCTs with large sample and multiple centers in future. Moreover, only two of the included studies reported the condition about adverse reaction. In view of the lack of information on this aspect, more relevant studies reporting adverse reactions are needed in the future.

## Figures and Tables

**Table 1:** Overview for the general characteristics of the included studies.

Studies	Number		Age (year)	Disease course (year)	Treatment (week)	Intervention		Outcome indicators
	EG/CG	M/F				EG	CG	
Lv et al. 2006 [9]	48/32	44/36	EG: 49.5 ± 9.5 CG: 48.6 ± 8.9	/	16	JSB + Valsartan	Valsartan	UAER
Cao et al. 2007 [10]	30/30	28/32	EG: 59.4 ± 14.1 CG: 58.9 ± 11.2	EG: 8.4 ± 5.3 CG: 8.9 ± 5.0	24	JSB + Valsartan	Valsartan	UAER, ACR, SBP, DBP, HbA_1c_
Lei et al. 2009 [11]	47/45	56/36	Total: 59.8 ± 7.1	Total: 8.3 ± 3.03	24	JSB + Irbesartan	Irbesartan	ORR, UAER, Scr, FBG, SBP, DBP, HbA_1c_
Zhang 2010 [12]	40/40	42/38	EG: 54.2 ± 8.4 CG: 50.2 ± 10.5	EG: 0.3~10.3 CG: 0.2~10.8	8	JSB + Candesartan cilexetil	Candesartan cilexetil	ORR
Tang 2011 [13]	40/40	47/33	EG: 59.2 ± 3.8 CG: 57.7 ± 4.2	EG: 16 ± 3.6 CG: 17 ± 2.8	12	JSB + Telmisartan	Telmisartan	ORR, UAER, Scr, FBG, SBP, DBP, HbA_1c_
Guo and Yan 2012 [14]	30/30	/	/	/	20	JSB + Irbesartan	Irbesartan	SBP, DBP
Li et al. 2012 [15]	38/38	42/34	EG: 48.6 CG: 49.3	EG: 7.4 CG: 7.7	8	JSB + Irbesartan	Irbesartan	ORR, 24 h UTP
Zhang et al. 2012 [16]	30/30	31/29	/	/	12	JSB + Valsartan	Valsartan	UAER, ACR, HbA_1c_, TC, TG, SBP, DBP
Gao and Wei 2013 [17]	100/105	118/87	EG: 57.3 ± 5.7 CG: 59.3 ± 5.2	EG: 13.9 ± 4.5 CG: 14.5 ± 5.1	12	JSB + Valsartan	Valsartan	24 h UTP, BUN, Scr
Yang 2013 [18]	20/20	21/19	EG: 50.8 ± 7.39 CG: 49.1 ± 7.9	EG: 11.2 ± 5.3 CG: 10.7 ± 5.5	12	JSB + Losartan	Losartan	UAER, BUN, Scr, TC, TG, FBG, HbA_1c_
Yu et al. 2013 [19]	40/40	52/28	Total: 47.6 ± 3.2	Total: 11.5 ± 2.9	12	JSB + Olmesartan Medoxomil	Olmesartan Medoxomil	24 h UTP, ACR
Ding 2014 [20]	50/50	52/48	Total: 51.2 ± 9.5	/	16	JSB + Irbesartan	Irbesartan	24 h UTP, BUN, Scr
Xiang 2014 [21]	60/60	71/49	EG: 41~71 CG: 42~70	/	12	JSB + Olmesartan Medoxomil	Olmesartan Medoxomil	ORR, UAER, Scr, ACR
Zhang et al. 2014 [22]	30/30	/	Total: 64 ± 6.3	/	8	JSB + Candesartan cilexetil	Candesartan cilexetil	UAER, BUN, SCr, SBP, TC, TG, Cys-C, *β*_2_-MG
Zhang and Zuo 2014 (2) [23]	41/41	69/13	Total: 50.4 ± 10.2	/	8	JSB + Losartan potassium	Losartan potassium	UAER, SCr, Cys-C
Shen et al. 2015 [24]	30/30	29/31	EG: 52.5 ± 6.9 CG: 51.5 ± 6.5	EG: 3.5 ± 2.8 CG: 3.4 ± 2.7	24	JSB + Candesartan	Candesartan	UAER, ACR, SBP, DBP, HbA_1c_
Xu 2015 [25]	50/50	59/41	EG: 50.9 ± 5.1 CG: 52.1 ± 4.9	/	20	JSB + Irbesartan	Irbesartan	UAER, SBP, DBP
Zhu and Qiu 2015 [26]	30/30	31/29	EG: 40.9 ± 7.6 CG: 40.6 ± 7.5	/	20	JSB + Valsartan	Valsartan	24 h UTP, SBP, DBP
Dai 2016 [27]	45/45	64/26	EG: 52 ± 7.5 CG: 51 ± 7.5	EG: 10.3 ± 2.1 CG: 10.6 ± 1.5	28	JSB + Valsartan	Valsartan	24 h UTP, UAER, Scr, SBP, DBP, HbA_1c_
Pan and Shang 2016 [28]	40/40	52/28	EG: 64.5 ± 4.7 CG: 65.7 ± 5.2	EG: 12.3 ± 5.9 CG: 11.6 ± 6.3	12	JSB + Telmisartan	Telmisartan	ACR, BUN, Scr, SBP, DBP, TC, TG, *β*_2_-MG
Wu and Pan 2016 [29]	34/34	40/28	EG: 52.12 ± 3.23 CG: 50.56 ± 4.12	Total: 7.84 ± 2.12	8	JSB + Candesartan cilexetil	Candesartan cilexetil	ORR, UAER, BUN, Scr, SBP, DBP, Cys-C, *β*_2_-MG
Xiu 2016 [30]	55/55	54/56	EG: 53.2 ± 6.8 CG: 52.8 ± 5.7	EG: 0.4~8.5 CG: 0.5~8.5	12	JSB + Candesartan	Candesartan	UAER, SBP, DBP, HbA_1c_
Zhang 2016 [31]	33/34	33/34	EG: 56.4 ± 3.3 CG: 55.6 ± 4.1	EG: 5~10 CG: 6~9	12	JSB + Irbesartan	Irbesartan	UAER, HbA_1c_
Cai 2017 [32]	50/50	51/49	EG: 65.2 ± 1.3 CG: 65.5 ± 1.2	/	12	JSB + Olmesartan Medoxomil	Olmesartan Medoxomil	24 h UTP, ACR
Liu and Zhang 2017 [33]	41/41	43/39	Total: 63.3 ± 5.7	/	8	JSB + Valsartan	Valsartan	ORR, BUN, Scr, SBP, DBP, FBG, HbA_1c_, *β*_2_-MG
Zhu and Li 2017 [34]	53/53	62/44	EG: 52.9 ± 10.8 CG: 54.6 ± 10.4	EG: 8.9 ± 2.8 CG: 8.6 ± 2.5	8	JSB + Valsartan	Valsartan	UAER, Cys-C, *β*_2_-MG, SBP, DBP

EG, experimental group; CG, control group; M, male; F, female.
